# Guidelines for epilepsy management in India classification of seizures and epilepsy syndromes

**DOI:** 10.4103/0972-2327.74187

**Published:** 2010

**Authors:** Sridharan Ramaratnam, P Satishchandra

**Affiliations:** Department of Neurology, Apollo Hospitals, Chennai, India; 1 National Institute of Mental Health and Neurosciences, Bangalore, India

**Keywords:** Epilepsy, classification, seizures

## Abstract

This article is part of the Guidelines for Epilepsy management in India. This article reviews the classification systems used for epileptic seizures and epilepsy and present the recommendations based on current evidence. At present, epilepsy is classified according to seizure type and epilepsy syndrome using the universally accepted International League Against Epilepsy (ILAE) classification of epileptic seizures and epilepsy syndromes. A multi-axial classification system incorporating ictal phenomenology, seizure type, epilepsy syndrome, etiology and impairments is being developed by the ILAE task force. The need to consider age-related epilepsy syndromes is particularly important in children with epilepsy. The correct classification of seizure type and epilepsy syndrome helps the individual with epilepsy to receive appropriate investigations, treatment, and information about the likely prognosis.

## Introduction

Epilepsy is a symptom of an underlying neurological disorder and not a single disease entity. Hence, the classification of epilepsy evolved so far is not ideal. The first attempt to classify the epilepsies was carried out by Gastaut,[1] which formed the basis for the Commission on the Classification and Terminology of the International League against Epilepsy (ILAE) standardized classifications and terminology for epileptic seizures[2] and the epilepsies and epileptic syndromes[3] developed in the 1970s and 1980s.

## Classification of seizures

Seizures may be broadly classified as partial or generalized:

### 

#### Partial

- Begin focally in a restricted area of the cortex. This may be simple partial (without loss of awareness) or complex partial (loss of awareness). Simple partial seizures may present with motor, somato sensory, special sensory, psychic and autonomic.
- May spread and cause generalized tonic-clonic seizure

#### Generalized

- Believed to arise diffusely in both hemispheres;
- Bilateral (nonfocal) seizure onset;
- May present as absences, myoclonic seizures, tonic seizures, clonic seizures, tonic-clonic seizures or as atonic seizures

#### Unclassified

At present, the classification of the International League Against Epilepsy[2] (ILAE) remains the most widely used classification

## Classification of epilepsy

ILAE proposed a classification of the epilepsies[3] to better characterize them. This is similar to the classification of seizures. Two broad distinguishing features are used

- Between localization-related epilepsies, characterized by seizures that have a focal or partial onset, and generalized epilepsies, characterized by seizures with diffuse cortical origin. Some epilepsy syndromes may not be distinguishable as to whether generalized or localization related.
- Between idiopathic and symptomatic epilepsies
  - Symptomatic epilepsies are those associated with a known or suspected brain disease or lesion.
  - Seizures where an underlying brain insult appears likely, but whose causes cannot be identified are termed cryptogenic and are included in the symptomatic grouping.
  - Epilepsies that are inherited or are not symptomatic without identifiable pathologic cause are labeled as idiopathic.

Although the ILAE 1981 and 1989 classifications remain in common use they have been the subject of criticism and debate. They have been criticized for:

- being unsatisfactory for epidemiological research
- placing undue emphasis on the types of case referred to tertiary centers
- placing undue emphasis on the role of the EEG at the expense of newer techniques such as MRI
- not classifying epileptic seizures according to what an individual or eyewitness reports happens during a seizure (ictal semiology).

## Semiological classification

A semiological classification of epilepsy based on ictal events has been proposed by Luders and co workers.[4]

## Multiaxial classification

The ILAE in 1997 made revision of classification a priority and set up a Task Force of experts in the field to address this issue. The Task Force proposed that clinicians and researchers should use a multi-axial diagnostic scheme.[5] Epileptic seizures and epilepsy syndromes should be described and categorized according to a system that uses standardized terminology, and that is sufficiently flexible to take into account the following aspects of epilepsy diagnosis:

1. Some individuals cannot be given a recognized syndromic diagnosis;
2. Seizure types and syndromes change as new information is obtained;
3. Complete and detailed descriptions of ictal phenomenology are not always available.

Multi axial classification scheme has been designed to suit specific purposes (for example, communication and teaching; therapeutic trials; epidemiologic studies; selection of patients for epilepsy surgery; basic research; genetic studies). The classification system can be simplified or expanded, depending on whether it has to be used by a neurologist with particular expertise in epilepsy or by a general physician or pediatrician. The specific areas covered by this scheme[5] are:

Axis 1: *Ictal phenomenology*, from the Glossary of Descriptive Ictal Terminology to describe ictal events.

Axis 2: *Seizure type*, from the List of Epileptic Seizures - Localization within the brain and precipitating stimuli for reflex seizures should be specified when appropriate.

Axis 3: *Syndrome*, from the List of Epilepsy Syndromes, though a syndromic diagnosis may not always be possible.

Axis 4: *Etiology*, from a Classification of Diseases Frequently Associated with Epileptic Seizures or Epilepsy Syndromes when possible, genetic defects, or specific pathologies causing symptomatic focal epilepsies.

Axis 5: *Impairment*, this optional, but often useful, additional diagnostic parameter can be derived from an universally accepted impairment classification. The Task Force also made suggestions as to how current terminology should be changed so as to make it more usable.

For the full classification, the reader may refer to the references provided.

## Literature base regarding importance of classification

Several authors[6–9] have emphasized the importance of making the correct diagnosis and classification especially in juvenile myoclonic epilepsy (JME), which is often, misdiagnosed resulting in uncontrolled seizures. Once a correct diagnosis of JME has been made, the majority of subjects who have been uncontrolled earlier become seizure free or well controlled with valproate or other drugs.[6–8] These authors suggested that a syndromic classification should be recorded for all people with epilepsy, and this should be regularly reviewed if seizures are poorly controlled. The most frequent reason for misdiagnosis was an underestimation or misinterpretation of myoclonic jerks suggesting that the correct diagnosis is dependent on the knowledge of the physician. Another factor associated with misdiagnosis was a failure to seek a history of myoclonic jerks, again associated with the knowledge of the referring physician of the syndrome.[9]

Evidence statements: [Table 1 for levels of evidence]

**Table 1:** Level of evidence

Systematic review or meta-analysis of randomized controlled trials or at least one randomized controlled trial At least one well-designed controlled study without randomization or well designed cohort study Well-designed non-experimental descriptive studies, casecontrol studies, and case series Expert opinion

The classification of epilepsy is based on expert committee reports (International League Against Epilepsy). The present classification system is undergoing revision.[Table 1, iv]

Failure to correctly classify the epilepsy syndrome can lead to inappropriate treatment and persistence of seizures. [Table 1, iii]

Recommendations: [Table 2 for grades of recommendation]

**Table 2:** Grades of recommendation

A Based on level I evidence B Based on level II evidence or extrapolated from level I evidence C Based on level III evidence or extrapolated from level I or level II evidence D Based on level IV evidence or extrapolated from level I, level II, or level III evidence GPP Good practice point based on the clinical experience of the guidelines developing team

Epileptic seizures and epilepsy syndromes in individuals should be classified using internationally accepted classification system [Table 2, D]

The description of seizure (ictal phenomenology), seizure type, syndrome, etiology and comorbidities should be determined for appropriate treatment and control of seizures and improvement of quality of life. [Table 2, D]

Individuals with epilepsy should be given information about their seizure type(s) and epilepsy syndrome, and the likely prognosis. [Table 2, GPP]
